# Region- and Layer-Specific Glutamatergic Synapse Development in the Nascent Cortical Hierarchy

**DOI:** 10.1523/JNEUROSCI.0293-26.2026

**Published:** 2026-07-03

**Authors:** Luca Discepolo, James McAllister, Rosie Russell, Sarah Apilado, Gabriella Margetts-Smith, Daniela Franchini, Seth G. N. Grant, Cian O’Donnell, Michael C. Ashby, Paul G. Anastasiades

**Affiliations:** ^1^Translational Health Sciences, University of Bristol, Bristol BS1 3NY, United Kingdom; ^2^Intelligent Systems Research Centre, Ulster University, Derry~Londonderry BT48 7JL, United Kingdom; ^3^School of Psychology & Neuroscience, University of Bristol, University Walk, Bristol BS8 1TD, United Kingdom; ^4^University of Exeter, Exeter EX4 4QH, United Kingdom; ^5^Genes to Cognition Programme, Institute for Neuroscience and Cardiovascular Research, University of Edinburgh, Edinburgh EH16 4SB, United Kingdom; ^6^Simons Initiative for the Developing Brain (SIDB), Centre for Discovery Brain Sciences, University of Edinburgh, Edinburgh EH8 9XD, United Kingdom

**Keywords:** developmental biology, layer, neocortex, prefrontal, somatosensory, synapse

## Abstract

Neocortical synapses are highly dynamic during brain development, undergoing formation, elimination, and maturation before acquiring properties that support adult cognition. Individual neocortical regions develop at different ages, and individual layers within these regions contain distinct neuronal subtypes that process unique patterns of local and long-range synaptic input. To better understand the development of the cortical hierarchy, we explored the laminar maturation of glutamatergic synapses across cortical regions of male and female mice. Synapse maturation was associated with the upregulation of the postsynaptic density protein PSD95. This maturation occurred in a region- and layer-specific manner—layers associated with feedforward pathways develop earlier, while layers associated with higher-order circuits develop later. Our findings highlight adolescence as an important period for the cortex-wide maturation of synapses in cortical layer 1, synapses known to receive top–down feedback from higher-order cortices. We propose that this delayed adolescent maturation of top–down input represents a global signature of cortical development and seemingly acts as the final stage of outside–in brain maturation.

## Significance Statement

Our findings provide a high-throughput analysis of the postnatal development of glutamatergic synaptic proteins across the neocortical hierarchy. We highlight key differences in the maturation of higher cognitive areas and sensory–motor areas. We also observe the delayed and protracted maturation of cortical layer (L)1, which develops in a neocortex-wide manner during adolescence. Using analysis of synaptic puncta and computational modeling, we explore the synaptic mechanisms that underlie these changes. This work highlights adolescence as an important period for the maturation of top–down inputs to L1, which may play an important role in the emergence of adult-like cognition during this developmental stage.

## Introduction

The neocortex is organized hierarchically, with sensory regions at the bottom and association cortices at the top (Felleman and Van Essen, 1991). The canonical view is that brain development mirrors this hierarchy, with sensory regions maturing prior to association areas across species (Reh et al., 2020; Larsen et al., 2023). However, evidence from primates and rodents challenges this idea, suggesting the maturation of sensory and association areas is remarkably similar (Bourgeois et al., 1994; Myme et al., 2003). Cortical maturation has predominantly been studied in primary sensory circuits due to the ease with which sensory afferents can be manipulated (Fox and Wong, 2005). These studies indicate that development follows the canonical cortical microcircuit (Douglas and Martin, 2004; Harris and Shepherd, 2015), with sensory critical periods occurring first in thalamocortical synapses and then within layer (L)4, followed by the ascending L4→L2/3 pathway and L2/3 recurrent synapses (Erzurumlu and Gaspar, 2012). This sequential maturation is termed “outside–in,” whereby critical periods occur along the arc of sensory information transfer from the periphery to the neocortex (Sehara and Kawasaki, 2011; Erzurumlu and Gaspar, 2012). If, or how, layer-specific maturation occurs in higher-order regions remains to be determined.

Brain development culminates in adolescence when higher-order brain regions, including the prefrontal cortex (PFC), undergo final maturation (Larsen and Luna, 2018; Delevich et al., 2018; Anastasiades et al., 2022). This process supports the acquisition of adult-like cognition (Larsen and Luna, 2018; Zhu et al., 2024) and likely contributes to the increased susceptibility of the adolescent brain to neuropsychiatric and mental health conditions (Blakemore, 2019; Solmi et al., 2021). Despite its clear importance, less is known about PFC maturation. PFC thalamic input is present from the first postnatal week in rodents (Ferguson and Gao, 2014). Neural activity during this time is important for network formation and function (Bitzenhofer et al., 2021) and depends on preconfigured synaptic parameters (Chini et al., 2024). Subsequent thalamic activity orchestrates adult-like connectivity and cognition (Benoit et al., 2022; Petersen et al., 2024; Yang et al., 2025). However, it is unclear if the PFC undergoes thalamus-first maturation, mirroring sensory cortices, or if its distinct architecture (Anastasiades and Carter, 2021) gives rise to alternative layer- or input-specific development (Pattwell et al., 2016; Kroon et al., 2019).

The laminar separation of neocortical inputs is evident in the feedforward and feedback projections that mediate communication between higher-order association and lower-order sensory regions, with feedforward inputs targeting middle layers and feedback connections innervating L1 (Felleman and Van Essen, 1991; Harris et al., 2019). Cortical L1 displays significant complexity, acting as a node for the integration of long-range inputs (Huang et al., 2024), which account for ∼90% of L1 synapses (Larkum, 2013). These inputs convey predictions and contextual information and signal behavioral outcomes to support goal-directed behavior (Norman et al., 2021; Pardi et al., 2023). Despite L1’s importance, studies of cortical development have focused on feedforward pathways (Erzurumlu and Gaspar, 2012; Hooks and Chen, 2020). Consequently, it remains unclear if improvements in cognitive capabilities that depend on feedback are driven by the maturation of frontal cortices themselves (Nabel et al., 2020) or if sensory areas undergo protracted development to better respond to feedback.

Long-range cortical communication is mediated by glutamatergic synapses, whose number, morphology, and molecular composition undergo significant maturation (Lohmann and Kessels, 2014), contributing to the formation of new memories and the emergence of higher cognitive functions (Migaud et al., 1998; Fitzgerald et al., 2015). Cortical synapses are dynamic across development (Bhatt et al., 2009; Holtmaat and Svoboda, 2009), displaying an initial phase of overproduction followed by refinement (Bourgeois et al., 1994; Grutzendler et al., 2002; Y. Zuo et al., 2005; Johnson et al., 2016; Nagappan-Chettiar et al., 2024). At the molecular level, synapse maturation involves transitions in glutamate receptors (Ashby and Isaac, 2011; Matta et al., 2011; Anastasiades and Butt, 2012; Frank et al., 2016; Skene et al., 2017) and scaffold proteins that help organize the postsynaptic density (PSD; Sheng and Kim, 2011). The membrane-associated guanylate kinase (MAGUK) proteins PSD95 and SAP102 are key components of the PSD (Husi et al., 2000; Sheng and Kim, 2011; Zheng et al., 2011). Individual synapses contain unique combinations of PSD95 and SAP102 (Zhu et al., 2018), which contributes to region- and layer-specific synaptic function (Hansen et al., 2025). The expression of PSD95 and SAP102 is highly developmentally regulated (Cizeron et al., 2020), yet it is unclear if, or how, this process contributes to the development of layers and regions across the cortical hierarchy.

This study aims to determine synaptic maturation across the cortical hierarchy: focusing on the primary somatosensory (barrel) cortex (SSp) and the prelimbic subdivision of the PFC. Using high-throughput automated detection of fluorescently tagged puncta alongside computational modeling, we highlight similarities and differences in the synaptic maturation of neocortical regions.

## Materials and Methods

### Experimental animals

Male PSD95^eGFP/eGFP^; SAP102^mKO2/y^ and female PSD95^eGFP/eGFP^; SAP102^mKO2/mKO2^ (Zhu et al., 2018) or C57BL/6J mice (Charles River Laboratories) were housed in our animal facility on a 12/12 h light/dark cycle with ad libitum access to food and water. For all experiments, the age range for each developmental cohort was ±2 d of the reported age. All animal experiments were performed in accordance with The Animal (Scientific Procedures) Act 1986.

### Histology and immunohistochemistry

Mice received a lethal dose of sodium pentobarbital (200 mg/ml) before cardiac perfusion with 0.01 M PBS followed by 4% PFA. The brain was then extracted and stored in 4% PFA at 4°C until slicing. Slicing was performed on a Leica VT1000S Vibratome. For PSD95 and SAP102 labeling, 50 µm (confocal) or 70 µm (widefield) sections were taken and mounted on Superfrost slides (Thermo Fisher Scientific) with VECTASHIELD Mounting Medium with DAPI (Vector Labs) and then coverslipped. For VGlut2 staining, slices were cut at 50 µm and underwent antigen retrieval in sodium citrate buffer at 80°C for 30 min. Slices were allowed to cool before being washed in 0.01 M PBS. Slices were incubated at room temperature for 1 h in blocking solution: 5% normal goat serum in PBS-T (0.01 M PBS and 0.3% Triton X-100) with 0.05% sodium azide. Slices were then incubated with guinea pig anti-Vglut2 (1:1,000; Synaptic Systems, catalog #135404) in blocking solution overnight at 4°C. Slices were washed in 0.01 M PBS before the secondary antibody (goat anti-guinea pig Alexa Fluor 647, 1:1,000; Invitrogen, A21450) was applied in blocking solution for 1 h at room temperature. Slices were washed in 0.01 M PBS and mounted on Superfrost slides using ProLong Gold mounting media with DAPI (Invitrogen) and then coverslipped.

### Imaging

All imaging was undertaken at the Wolfson Bioimaging Facility at the University of Bristol. Widefield images were obtained with an Olympus VS200 slide scanning microscope using a 10× (NA 0.4) objective. For region-specific analysis, confocal images were obtained on a Leica SP8 confocal microscope using either a 10× (NA 0.3) or 63× (NA 1.4) oil immersion objective. The confocal pinhole size was set at 1 Airy unit. Refractive index of immersion oil was 1.52 and refractive index of VECTASHIELD mounting media was 1.45. For puncta imaging across the depth of the cortex, we captured five separate images using the 63× objective. The top of Position 1 was aligned to the pial surface with 200 µm spacing between images. For PSD95 and SAP102 imaging, we calibrated microscope settings to allow reliable signal detection across the entire developmental range of the study in advance and applied the same settings across all imaging sessions.

### Puncta and nuclei detection

Puncta and nuclei detection was performed using the Modular Image Analysis (MIA) plugin for ImageJ (Cross et al., 2024). This first involved applying a 2D Gaussian filter (sigma = 2 px) to reduce noise, followed by the sliding paraboloid implementation of background subtraction using a radius of 2 px. Puncta were detected using the Laplacian of Gaussian spot detector in TrackMate (Tinevez et al., 2017; implemented via MIA) and 2D Gaussian profiles fit to each detected punctum to estimate sigma. Spot intensity was measured for all puncta by taking into consideration all pixels within 2 px of the object.

The same plugin was also used to segment and measure nuclei. This involved applying a 2D median filter (radius = 2 px) followed by application of the sliding paraboloid background subtraction, however, retaining the background image as the ongoing representation of the nuclei. Nuclei were detected using the StarDist plugin (Schmidt et al., 2018) on images scaled in *X* and *Y* by a factor of 0.3. These nuclear objects were then converted to a binarized representation, upscaled to the full resolution, and subject to a watershed transform to ensure all proximal objects were detected separately (Legland et al., 2016). Any nuclei smaller than 25 μm^2^ or with texture “correlation” metrics <0.4 were removed from further analysis (Haralick et al., 1973).

For optimal detection, we required parameters that had good sensitivity to detect puncta but also specificity to avoid false positives. To determine detection parameters that reliably identified puncta across all ages of our developmental study, we chose a random subsample of images spanning all age groups and manually scored identified puncta across a range of detection settings. We chose settings that gave high detection accuracy across all ages. For each individual punctum, the MIA plugin calculated values of mean and max intensity. The 2D Gaussian profiles fit to each detected punctum was taken as a proxy for size.

To control for differences in cell density across development and layers, we performed additional analysis of puncta density which controlled for the area taken up by the cell nucleus, which is devoid of PSD95 puncta. For each image, we subtracted the total area of the nuclear objects detected using the MIA plugin from the area of the image and used this area value to calculate a corrected puncta density for each image using the formula below:

$$\mathrm{Corrected}\;\mathrm{Puncta}\;\mathrm{Density}\;=\frac{\mathrm{Number}\;\mathrm{of}\;\mathrm{Puncta}}{\left(\mathrm{Imaging}\;\mathrm{Area}-\mathrm{Nuclear}\;\mathrm{Area}\right)}.$$

To determine how individual puncta properties contribute to overall PSD95 fluorescence values, we took the average 10× fluorescence intensity and compared it to a model based on the calculated puncta number, average intensity and size values on a mouse-by-mouse basis. We used relative maturation values (ranging from 0 to 1) for all calculations, allowing us to determine how a developmental change in the properties of individual puncta parameters contributed to a change in bulk fluorescence. The puncta model was as below:

WidefieldMaturation=PunctaNumber×MeanPunctaIntensity×PunctaSize.

To determine the influence of the different puncta parameters on PSD95 development, either puncta number, intensity, or size was artificially set to zero. The corresponding model error was calculated in each case on a mouse-by-mouse basis and pooled across developmental stage to determine the variable that most strongly impacted model performance.

### Computational modeling

Computational modeling was performed to simulate and analyze the dynamics of synapse formation, maturation, elimination, and synaptic weight. The model considered three possible states of a given synapse: a potential resource pool (*P*), an immature population (*I*), and a mature population (*M*). The numbers of synapses in the resource pool, immature population, and mature population are denoted by *N_P_*, *N_I_*, and *N_M_*, respectively. All synapses start in the potential resource pool but may transition from the resource pool to the immature state (“synapse creation”), from the immature state to the potential resource pool (“synapse elimination”), from the immature state to the mature state (“synapse maturation”), and from the mature state to the immature state (“synapse dematuration”). These transitions are regulated by the creation rate (*c*), elimination rate (*e*), maturation rate (*m*), and dematuration rate (*d*).

Population dynamics of synapses was modeled with two different approaches for comparison, using (1) random walks and (2) differential equations:

(1) Random walks

In the random walks version, each synapse is represented by a random variable that can occupy one of the three states *P*, *I*, and *M*. At each discrete time step, a synapse may transition between states based on specific probabilities *p_c_*, *p_e_*, *p_m_*, and *p_d_* (derived from the rates *c*, *e*, *m*, and *d*) and according to the transitions presented by the transition matrix *T*:

$$T=\left[\begin{array}{ccc} 1-p_{c} & \;p_{c} & 0\\ \;p_{e} & 1-(\;p_{e}+p_{m}) & \;p_{m}\\ 0 & \;p_{d} & 1-p_{d} \end{array}\right].$$

Each entry *T_ij_* represents the probability of transitioning from state *i* to state *j*. For example, the entry $T_{PI}(=T_{12})=p_{c}$ denotes the probability of a synapse transitioning from the potential resource pool state to the immature state. This transition matrix summarizes the fact that a synapse can transition *P* ⇌ *I* and *I* ⇌ *M* with relevant probabilities, but not directly *P* ⇌ *M*. At every timestep, the model calculates the numbers of synapses in each of the three states, giving results for *N_P_*, *N_I_*, and *N_M_* across time. This Markov chain model is simulated over many trials to calculate the average population dynamics.

The random walks model has the advantage that its stochastic nature captures the randomness inherent in biological processes, providing a relatively realistic simulation of individual synapse behavior. However, it is more computationally expensive and less analytically insightful.

(2) Differential equations

In the differential equations model, the population dynamics are described by the following equations:

$$\frac{dN_{P}}{dt}=e\;N_{I}-c\;N_{P},$$

$$\frac{dN_{I}}{dt}=c\;N_{P}-(e+m)\;N_{I}+d\;N_{M},$$

$$\frac{dN_{M}}{dt}=m\;N_{I}-d\;N_{M}.$$

The differential equations model loses the stochastic nature of the individual synapse state transitions, instead modeling average population behavior. However, it is more computationally efficient and lends itself more easily to analysis.

For all synapses in the mature population, we also model synaptic weight dynamics using a Kesten process, which has been previously found to account for the fluctuations of synapse sizes over time (Statman et al., 2014). This model is a combination of stochastic multiplicative and additive processes, described by the following equation:

$$w_{t+1}=\epsilon w_{t}+\eta ,$$

where *w_t_* represents the synaptic weight at time *t* and ϵ and *η* are the multiplicative and additive normal random variables, respectively. At every timestep, a random number is sampled independently for both ϵ and *η*, which together cause the synaptic strength to fluctuate stochastically over time. Parameter values were based on Statman et al. (2014). The simulation timestep was set to 0.1 d. The Kesten process with appropriate parameters gives resulting synaptic strength distributions that are broad, skewed, and relatively stable over time, yet individual synapse strengths exhibit significant spontaneous fluctuations (Statman et al., 2014). In the differential equations version where population dynamics are modeled instead of individual synapses, the model continues in parallel to stochastically simulate individual synapses within the mature population by the Kesten process, ensuring that the number within this simulated weight dynamics pool aligns with the population count prescribed by the differential equations.

The model was initially implemented with constant transition rates *c, m, e*, and *d*, with the only added rule that when a synapse transitions from the mature to immature population, the synapse with the smallest synaptic weight is removed from the mature population (this has no impact on the population dynamics). However, mathematical analysis (Text S1) proved that, if all synapses start in the resource pool, then the combined synapse population (*N_I_* + *N_M_*) increases strictly monotonically with constant values of *c*, *m*, *e*, and *d*. To capture an initial increase and then elimination of synapses, we proceeded to use variable rates with developmental age.

Variable rates of synapse creation, elimination, and dematuration are more neurobiologically realistic and give rise to more complex population dynamics. A variable rate model implemented the rates *c* and *e* in a time-dependent manner using exponential functions such that the rates start high and decrease to a baseline over developmental time (Fig. S9*B*). The equations are as follows:

$$c(t)=A_{1}e^{-\;\frac{t}{\tau_{1}}}+b_{1},$$

$$e(t)=A_{2}e^{-\;\frac{t}{\tau_{2}}}+b_{2}.$$

The parameter *b*_1_ controls the asymptotic baseline value that the rate decays to; *τ*_1_ is the time constant and controls the timescale of the exponential decay; *A*_1_ is the amplitude coefficient, determining the initial magnitude of the decaying component. In this study, the rates were set such that *b*_1_ = *b*_2_ = 0.2, ensuring that creation and elimination rates eventually equilibrate, consistent with experimental data (Holtmaat et al., 2005). We also modeled a variable rate of dematuration, however in a weight-dependent manner, for all synapses in the mature population. This can also be modeled simply with an exponential function as follows:

$$d(w)=A_{3}\;e^{-\;\frac{w}{\gamma }}.$$

Here *w* is the synaptic weight of a given synapse; *A*_3_ is the amplitude coefficient; and *γ* is the weight decay constant. This part of the model did not include a positive baseline *b* term, to ensure that the probability is vanishing for increasingly large synapses. This captures the observation that biologically, smaller synapses are more likely to demature and large synapses are unlikely to demature (Trachtenberg et al., 2002; Holtmaat et al., 2005). For the variable dematuration rate in the differential equations model, the model calculates *d* by taking a weighted expectation through the probability distribution across synaptic weights. Synapses are then removed probabilistically from the simulated weight dynamics population (for the Kesten process) to align with the population count *N_M_* computed by the differential equations. Values for model parameters are shown in Table 1.

**Table 1.** Computational model parameter values

Parameter	Symbol	Value
Kesten process additive noise mean	$\eta_{\mu }$	0.0077
Kesten process additive noise SD	$\eta_{\sigma }$	0.03
Kesten process multiplicative noise mean	$\epsilon_{\mu }$	0.9923
Kesten process multiplicative noise SD	$\epsilon_{\sigma }$	0.05
Creation rate amplitude, P0 offset	*A*_1_	0.9
Creation rate decay time constant	*τ*_1_	30 d
Creation rate amplitude, adulthood	*b*_1_	0.2/d
Elimination rate amplitude, P0 offset	*A*_2_	3.5/d
Elimination rate decay time constant	*τ*_2_	6 d
Elimination rate amplitude, adulthood	*b*_2_	0.2/d
Maturation rate	*m*	0.05/d
Dematuration rate, zero-strength synapses	*A*_3_	0.5/d
Dematuration weight decay constant	*γ*	0.6

We performed a global sensitivity analysis on the model to quantify how variations in the rate parameters (*A*_1_, *τ*_1_, *A*_2_, *τ*_2_, *m*, *A*_3_, *γ*) affected the final synapse counts and the timing of peak synapse numbers. We used the regression method in the Julia package GlobalSensitivity.jl to perform this analysis, which produced standard regression coefficients for each parameter and outcome of interest.

We calculated the survival fraction of synapses during two developmental stages: early development (PND 16–26) and adulthood (PND 70–88), corresponding with empirical data in Holtmaat et al. (2005). To compute the survival fractions, we took the initial synapse count in the combined population at the beginning of each of these developmental stages and then tracked the states of these synapses, calculating the fraction that survive over the subsequent days relative to the initial count. This was repeated over multiple trials and averaged.

### Layer assignment in confocal images

**Figure 1.**
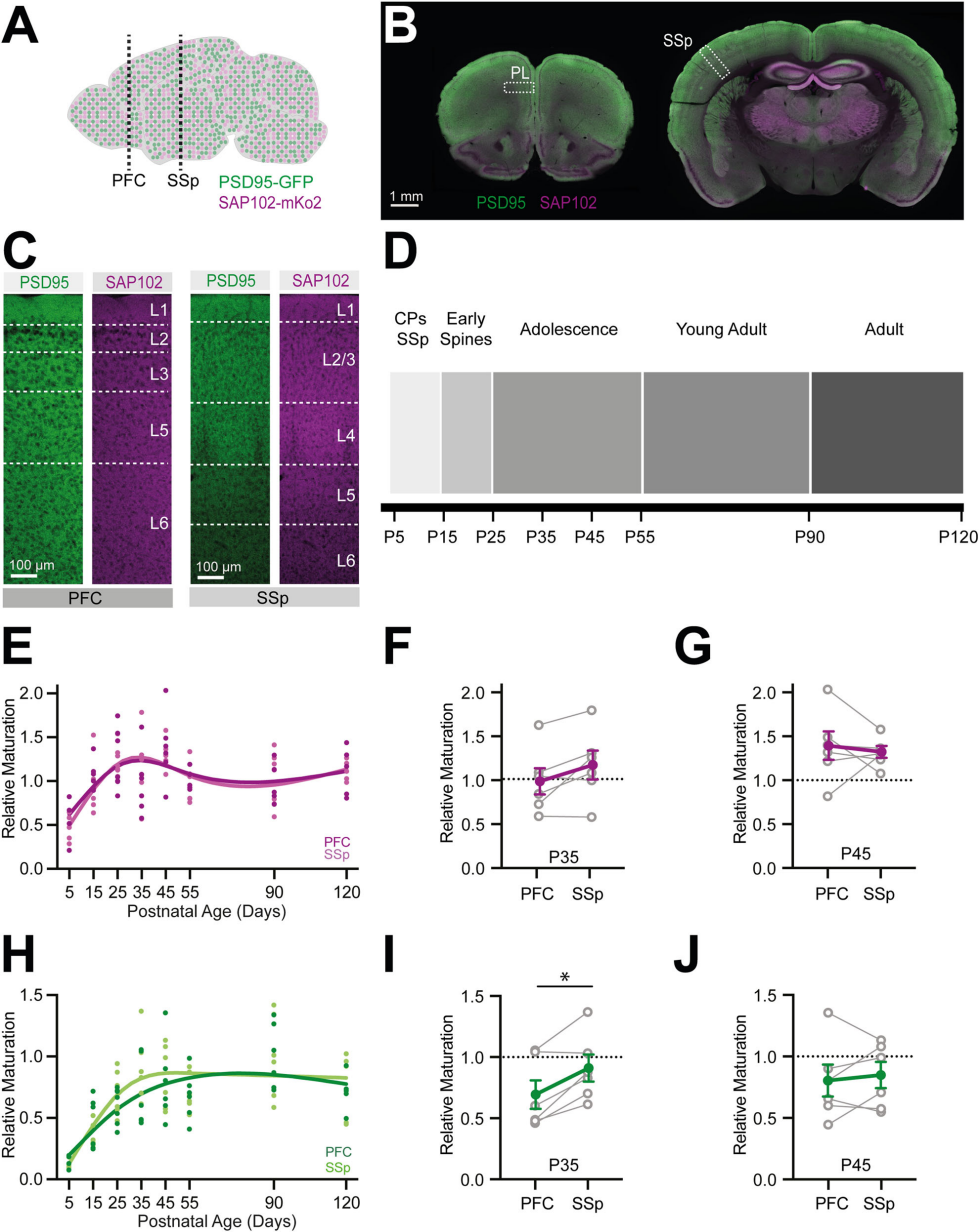
***A***, Schematic showing transgenic mouse line and regions of interest. ***B***, Example coronal sections showing the regions of analysis in the prelimbic (PL) PFC (left) and somatosensory barrel cortex (SSp; right). ***C***, Example images showing the distribution of PSD95 and SAP102 labeling across the laminar depth of the PFC (left) and SSp (right). ***D***, Developmental timeline showing key periods in the maturation of the neocortex. CPs = critical periods. ***E***, Relative maturation of average SAP102 expression in PFC and SSp normalized to adult levels (P90). Points represent individual mice with fit line to the individual data points. ***F***, Relative SAP102 maturation in PFC and SSp at P35 showing pairwise, within-mouse comparisons. *p* = 0.08. ***G***, Relative SAP102 maturation in PFC and SSp at P45 showing pairwise, within-mouse comparisons. *p* = 0.6. ***H–J***, As ***E–G*** but for PSD95. Panel ***I***, *p* = 0.01; panel ***J***, *p* = 0.6. Number of mice per age group: PFC, P5 = 5, P15 = 6, P25 = 7, P35 = 6, P45 = 6, P55 = 6, P90 = 6, P120 = 5; SSp, P5 = 5, P15 = 6, P25 = 7, P35 = 6, P45 = 6, P55 = 6, P90 = 6, P120 = 5. Values are mean average ± SEM, with exception of data in ***E*** and ***H*** which show smoothing spline fit to the individual data points. *p < 0.05.

**Figure 2.**
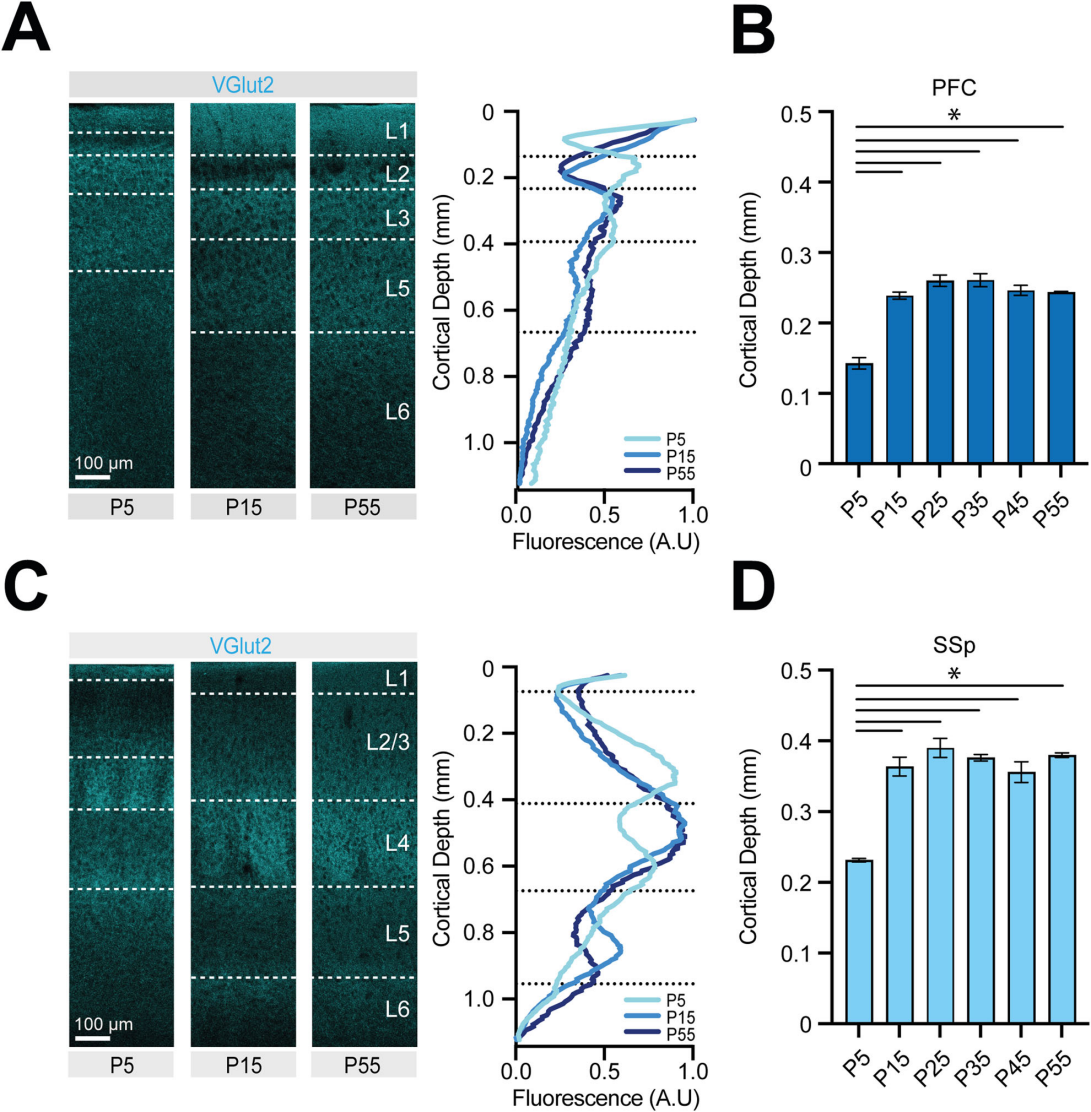
***A***, Left, Representative images of VGlut2 staining across PFC layers at P5, P15, and P55. Right, Average VGlut2 fluorescent profiles across all mice imaged at P5, P15, and P55 showing shift in distributions across development. Dashed lines indicate individual adult layer boundaries. ***B***, Quantification of the cortical depth of the second peak in VGlut2 labeling, corresponding to the location of L3 in the PFC (Collins et al., 2018). Data are shown at different postnatal ages spanning cortical development. *p* < 0.0001 for P5 and all other ages. ***C, D***, Similar to ***A*** and ***B*** but for SSp. Data in ***D*** calculate the cortical depth corresponding to the start of the L4 barrels. *p* < 0.0001 for P5 and all other ages. Values are shown as mean average ± SEM, with exception of plots in ***A*** and ***C*** where error bars have been omitted for clarity. *p < 0.05.

**Figure 3.**
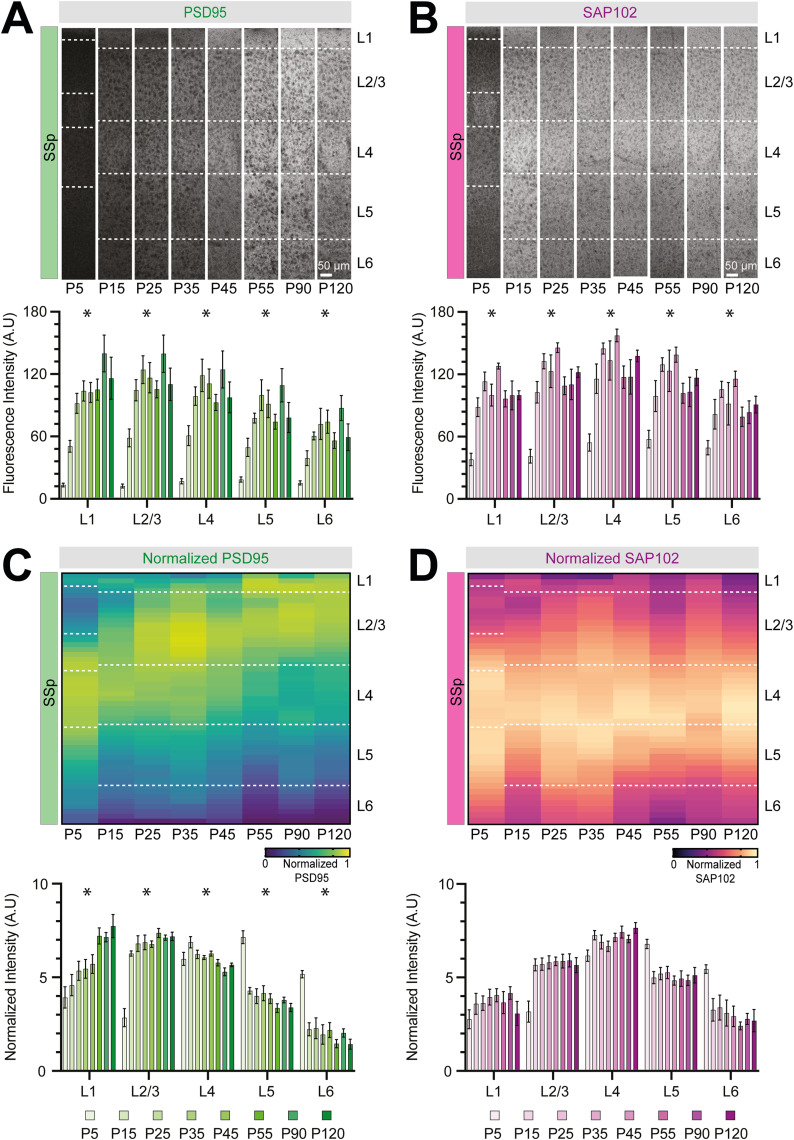
***A***, Top, Representative images showing the distribution of PSD95 across SSp layers at different postnatal ages. Bottom, Quantification of raw PSD95 fluorescence values for each layer of SSp across development. Layer *p* ≤ 0.0001; age *p* ≤ 0.0001. ***B***, Top, Representative images showing the distribution of SAP102 across SSp layers at different postnatal ages. Bottom, Quantification of raw SAP102 fluorescence values for each layer of SSp across development. Layer *p* ≤ 0.0001; age *p* ≤ 0.0001. ***C***, *Top*, Heat maps showing the average peak normalized distributions of PSD95 across SSp layers at different postnatal ages. Bottom, Quantification of normalized PSD95 fluorescence density for each layer of SSp across development. Layer *p* ≤ 0.0001; age *p* = 0.028. ***D***, Top, Heat maps showing the average peak normalized distributions of SAP102 across SSp layers at different postnatal ages. Bottom, Quantification of normalized SAP102 fluorescence density for each layer of SSp across development. Layer *p* ≤ 0.0001; age *p* = 0.79. Number of mice per age group: SSp, P5 = 5, P15 = 6, P25 = 7, P35 = 6, P45 = 6, P55 = 6, P90 = 6, P120 = 5. All values are shown mean ± SEM. *, layers showing a significant change across development (*p* < 0.05).

**Figure 4.**
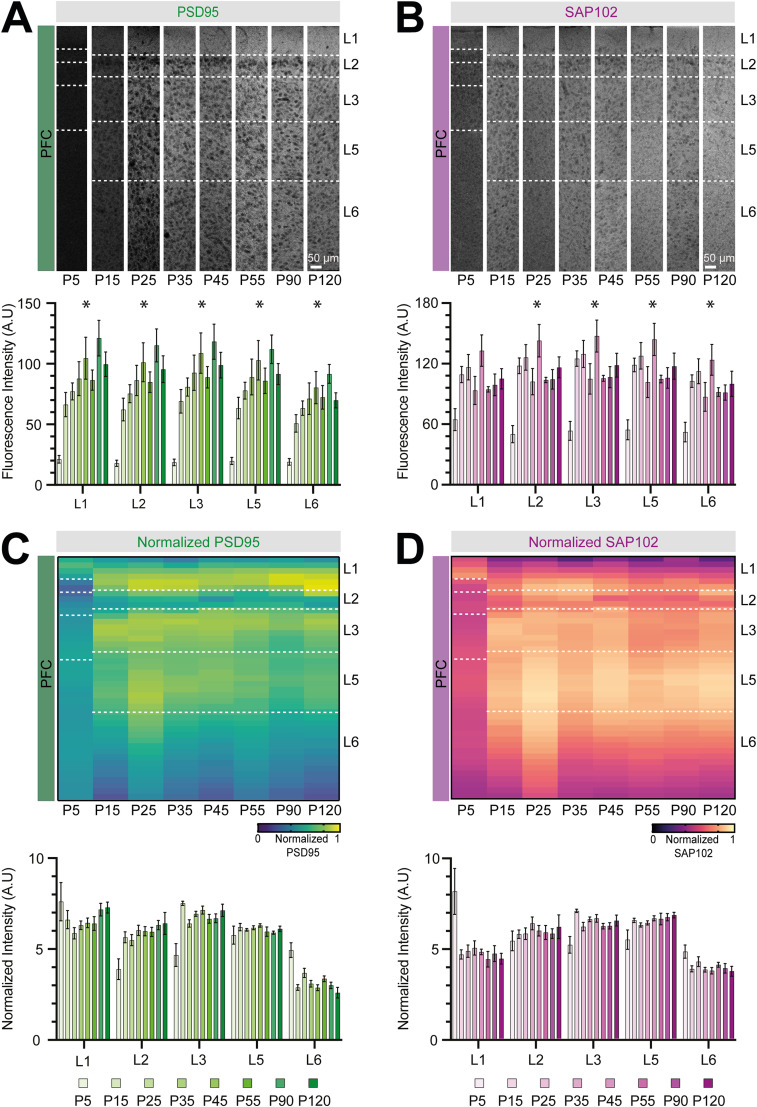
***A***, Top, Representative images showing the distribution of PSD95 across PFC layers at different postnatal ages. Bottom, Quantification of raw PSD95 fluorescence values for each layer of PFC across development. Layer *p* ≤ 0.0001; age *p* ≤ 0.0001. ***B***, Top, Representative images showing the distribution of SAP102 across PFC layers at different postnatal ages. Bottom, Quantification of raw SAP102 fluorescence values for each layer of PFC across development. Layer *p* ≤ 0.0001; age *p* = 0.001. ***C***, Top, Heat maps showing the average peak normalized distributions of PSD95 across PFC layers at different postnatal ages. Bottom, Quantification of normalized PSD95 fluorescence density for each layer of PFC across development. Layer *p* ≤ 0.0001; age *p* = 0.28. ***D***, Top, Heat maps showing the average peak normalized distributions of SAP102 across PFC layers at different postnatal ages. Bottom, Quantification of normalized SAP102 fluorescence density for each layer of PFC across development. Layer *p* ≤ 0.0001; age *p* = 0.75. Number of mice per age group: PFC, P5 = 5, P15 = 6, P25 = 7, P35 = 6, P45 = 6, P55 = 6, P90 = 6, P120 = 5. All values are mean ± SEM. *, layers showing a significant change across development (*p* < 0.05).

For developmental layer assignment from individual images of PFC and SSp (Figs. 1–4 and associated supplemental figures), we used a combination of VGlut2 staining and DAPI labeling combined with previous studies. VGlut2 staining was performed at Postnatal Day (P)5, P15, P25, P35, P45, and P55. At each age, measurements were made across multiple slices and animals to create average layer boundaries in each brain region. Given no changes in cytoarchitecture were observed post P15, we used a single set of layer boundaries for each region thereafter. In PFC, for P15 onward, we used layer assignment based on previous analysis of the distribution of thalamic inputs and projection neurons (Anastasiades et al., 2019), which strongly aligned with our VGlut2 data. At P5, the layer boundaries were determined based on average values of measurements taken across the DAPI and VGlut2 images, with layers scaled from the adult data to account for observed changes in labeling. For SSp, the L1/2 border was determined based on changes in cell density, the start and end of L4 based on the presence of barrels, and the L5/6 border based on the location of the second peak of VGlut2 labeling in deep layers. From P15 onward, we used a single set of layer boundaries in SSp based on the grand average of the P15 to P55 data.

### Brain atlas alignment

**Figure 5.**
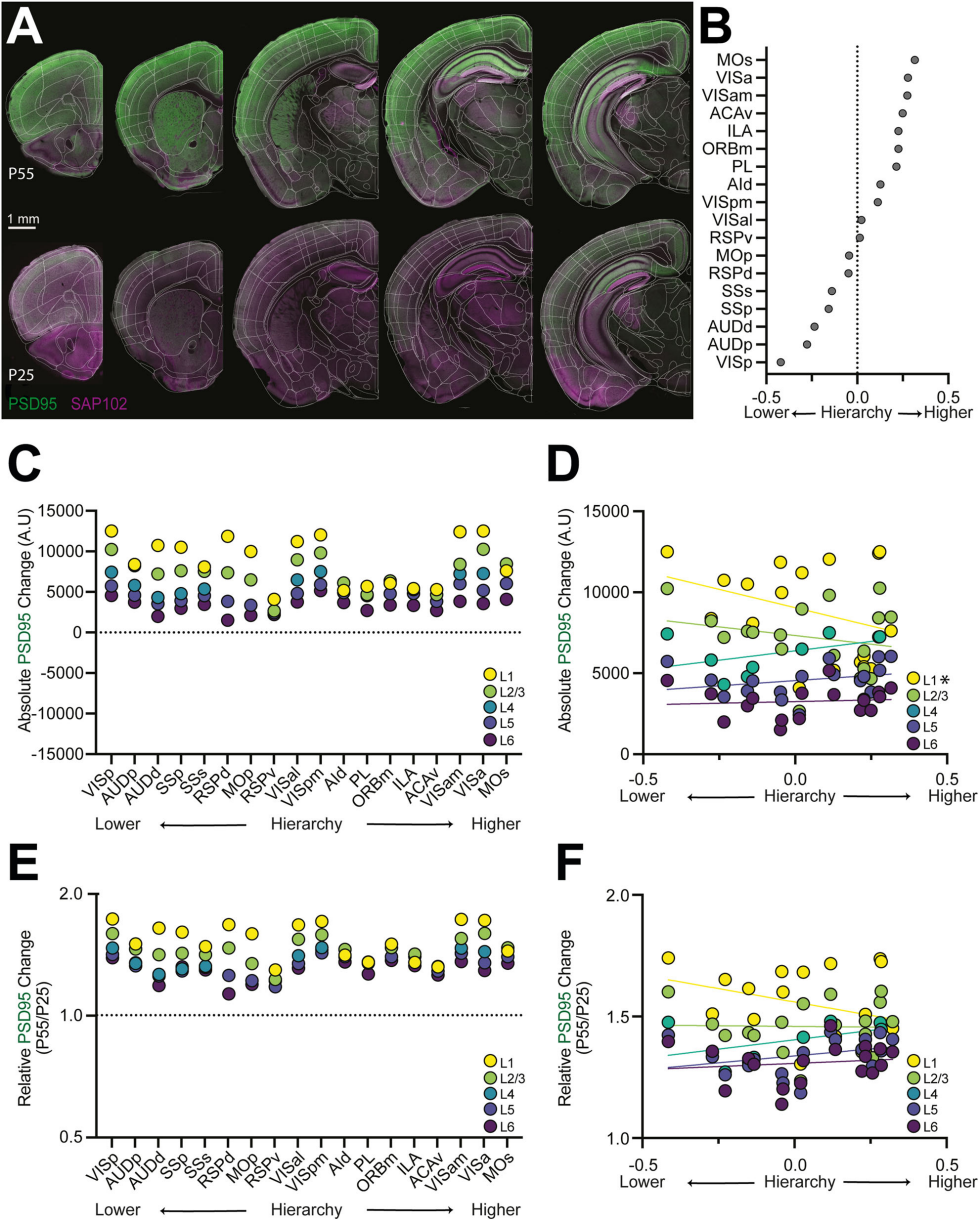
***A***, Representative images showing PSD95 (green) and SAP102 (magenta) labeling across different mouse brain slices spanning the cortical hierarchy at P55 (top) and P25 (bottom). Overlay shows the Allen Brain Atlas. ***B***, The hierarchy score for the different analyzed cortical regions based on cortical and thalamic connectivity (CC + TC + CT) from Harris et al. (2019). ***C***, Absolute adolescent change in PSD95 fluorescence levels calculated by subtracting the P55 values by the P25 values. Data points show average change for individual layers across the cortical regions shown in ***B***. ***D***, Correlation of the absolute adolescent change in PSD95 expression with the hierarchy position of each cortical area. Data are shown for each cortical layer for each of the regions shown in ***B***. L1 *p* = 0.04; L2/3 *p* = 0.2; L4 *p* = 0.2; L5 *p* = 0.3; L6 *p* = 0.7. ***E***, Relative adolescent change in PSD95 fluorescence levels calculated by dividing the P55 values by the P25 values. Data points show average change for individual layers across the cortical regions shown in ***B***. ***F***, Correlation of the relative adolescent change in PSD95 expression with the hierarchy position of each cortical area. Data are shown for each cortical layer for each of the regions shown in ***B***. L1 *p* = 0.09; L2/3 *p* = 0.9; L4 *p* = 0.17; L5 *p* = 0.18; L6 *p* = 0.56. The number of mice per group: P25 = 9, P55 = 11. Values are shown as mean average. SEM are omitted for clarity. *p < 0.05.

**Figure 6.**
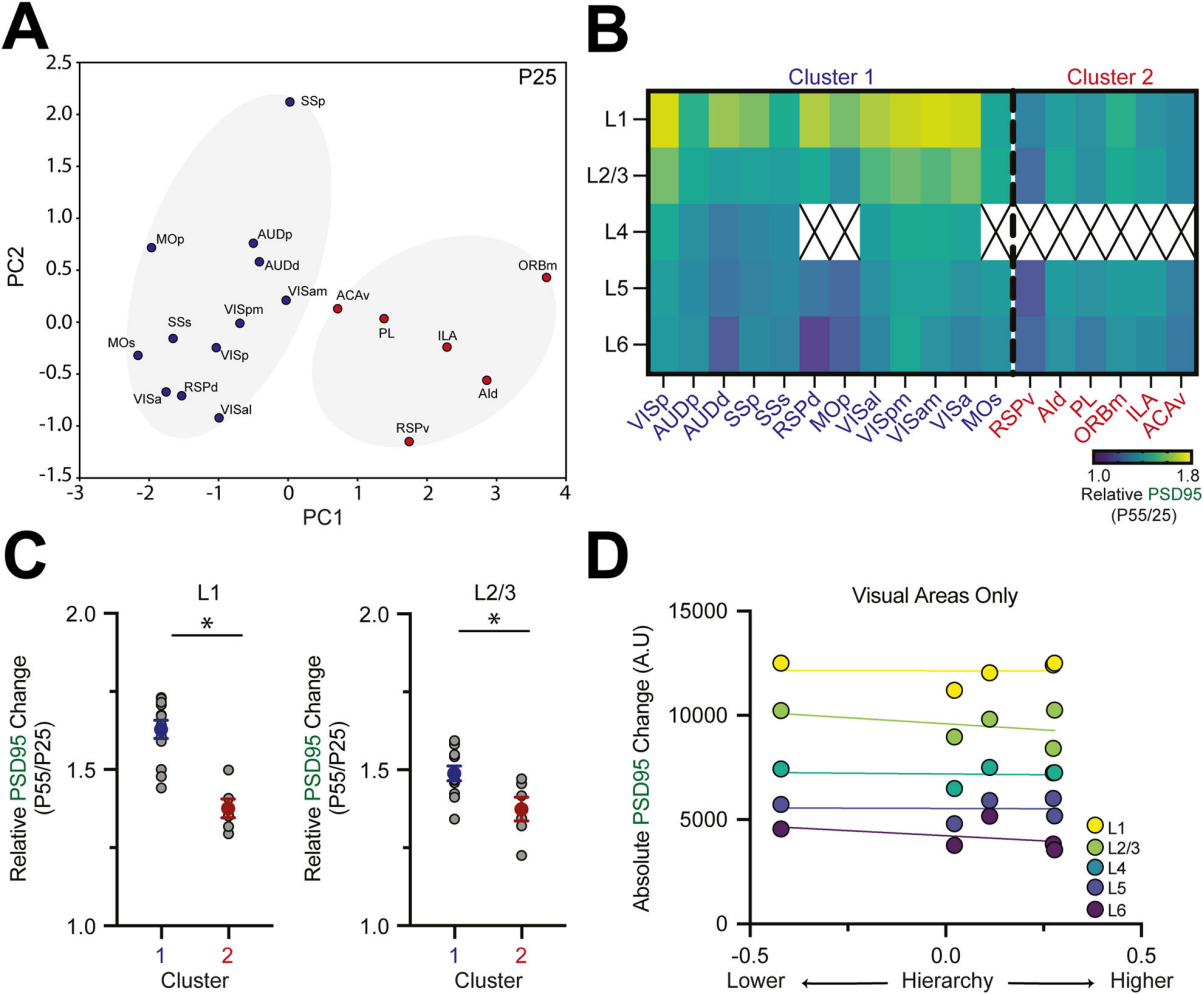
***A***, Principal component plot highlighting two clusters based on raw PSD95 expression levels at P25. Cluster 1 (blue) contains primarily sensory–motor regions, and Cluster 2 (red) contains mostly higher-order association cortices. ***B***, Heatmap showing the relative adolescent PSD95 change by layer and regions sorted based on the two clusters in ***A***. X indicates agranular cortex lacking L4. ***C***, Comparison of the relative adolescent change in L1 (left) and L2/3 (right) PSD95 signal between Cluster 1 and Cluster 2. L1 *p* ≤ 0.0001; L2/3 *p* = 0.018. ***D***, Correlation of the relative adolescent change in PSD95 expression with the hierarchy position of individual visual cortical areas. L1 *p* = 0.99; L2/3 *p* = 0.63; L4 *p* = 0.98; L5 *p* = 0.98; L6 *p* = 0.54. Number of brain regions per group: Cluster 1 = 12; Cluster 2 = 6. Values are shown as mean + SEM. SEM are omitted in ***B*** and ***D*** for clarity. *p < 0.05.

To examine the effect of hierarchical organization on the adolescent maturation of the cortex (Figs. 5, 6 and associated supplemental figures), we selected 18 cortical areas spanning the cortical hierarchy. The cortices analyzed were the secondary motor (MOs), prelimbic (PL), infralimbic (ILA), medial orbital (ORBm), anterior cingulate area ventral (ACAv), agranular insular area dorsal (AId), primary motor (MOp), primary somatosensory (SSp, focusing on SSpbfd), secondary somatosensory (SSs), ventral retrosplenial (RSPv), dorsal retrosplenial (RSPd), anteriomedial visual (VISam), anterior visual (VISa), dorsal auditory (AUDd), primary auditory (AUDp), posteriormedial visual (VISpm), primary visual (VISp), and anteriolateral visual (VISal). We imaged these areas at the start (P25) and end (P55) of adolescence. Hierarchical location was determined using the corticocortical, corticothalamic, and thalamocortical (CC + CT + TC) hierarchical connectivity scores from the Allen Connectivity Atlas (Harris et al., 2019).

Fluorescent profiles of the 18 cortical areas were obtained in Fiji with the BAR plugin (Ferreira et al., 2015) and background subtracted using off-slice pixel intensity. Profile measurements were recorded from both hemisphere across three slices per brain region. Cortical layers were defined using the ABBA (Aligning Big Brain Atlases) workflow aligning coronal sections to the Allen Mouse Atlas V3p1. Brain alignment was achieved in ABBA (Chiaruttini et al., 2025), uploaded to DeepSlice to register each slice to a comparable atlas section (Carey et al., 2023), with fine adjustments to account for dorsal–ventral and medial–lateral slicing angles performed in QuickNII (Puchades et al., 2019). Annotations were then exported to QuPath (Bankhead et al., 2017) where the registered atlas is overlayed over each image. Layer depths based on the atlas annotations were used to segment fluorescent profiles for relative comparison of average PSD95 and SAP102 fluorescence profiles using custom code in Python.

### Data analysis and statistics

Data are reported as mean averages ± SEM, unless otherwise stated. Comparisons between paired data in individual brain regions or mice were performed using a paired *t* test. Developmental comparisons were performed using two-way ANOVA with Tukey’s multiple-comparison correction. Comparisons between multiple groups were performed with one-way ANOVA or mixed-effects models for regional data with missing values in L4 or the Friedman test with correction for multiple comparisons. *K*-means clustering was performed in Python 3. Spline fitting was performed using a smoothing spline in Prism 10. Statistical analysis was performed in Prism 10 or Python 3. Computational modeling and simulations were performed using the Julia programming language (version 1.11.1).

## Results

### Postnatal development of MAGUK proteins across cortical regions

To study the synaptic maturation of the neocortex in a region- and layer-specific manner, we employed a transgenic mouse line which reports the endogenous expression of MAGUK proteins PSD95 and SAP102 by genetically tagging them with the fluorescent proteins eGFP and mKO2, respectively (Zhu et al., 2018). We focused our analysis on SSp and the PFC, allowing us to directly compare the maturation of a primary sensory area and a higher-order cognitive area (Fig. 1*A*–*C*). We imaged each area in animals aged P5 until 4 months (P120), a timespan encompassing multiple developmental stages (Fig. 1*D*). To compare how the expression of PSD95 and SAP102 changes across development, we imaged brain slices and calculated the average fluorescence across the depth of the cortex. We subsequently normalized each value by those observed in the adult (P90) to give a relative maturation score, such that 1 represents the average adult expression level and 0 represents a failure to detect meaningful levels of PSD95 or SAP102. This normalization allowed us to more easily compare the timeline of development for the MAGUK proteins, within and between areas. At P5, the relative maturation of SAP102 was significantly higher than PSD95 across both regions (Fig. S1). SAP102 levels continued to increase, peaking during adolescence before stabilizing in adulthood (Fig. 1*E–G*). In contrast, PSD95 expression increased steadily throughout development, reaching stable values by P55 (Fig. 1*H*–*J*) toward the end of adolescence. The ratio of SAP102/PSD95 expression reduced considerably in both regions as development progressed (Fig. S1), consistent with developmental shifts in SAP102 and PSD95 levels in the maturing brain (Zheng et al., 2011).

Although there was some variability in the fluorescence values between mice at any given age [see also Cizeron et al. (2020) ], there was strong intramouse correlation in regional fluorescence levels (Fig. S1). To establish if the two areas matured at different rates, we compared the relative fluorescence in SSp and PFC within individual mice. For SAP102, the relative maturation during adolescence was similar across both brain areas at both P35 (SAP102 P35, PFC 0.98 ± 0.1; SSp 1.16 ± 0.2; *p* = 0.08) and P45 (SAP102 P45, PFC 1.38 ± 0.2; SSp 1.31 ± 0.1; *p* = 0.6). This was not the case for PSD95, with the relative maturation during midadolescence (P35) significantly higher in SSp than PFC, indicating an earlier developmental timeline in somatosensory versus prefrontal areas (Fig. 1*I*; PSD95 P35, PFC 0.69 ± 0.1; SSp 0.90 ± 0.1; *p* = 0.01). This difference had disappeared at P45 during late adolescence (Fig. 1*J*; PSD95 P45, PFC 0.8 ± 0.1; SSp 0.84 ± 0.1; *p* = 0.6). These data indicate that PSD95 maturation is delayed in PFC compared with SSp.

### Defining cortical layers across development

To study layer-specific maturation, it was first necessary to determine how the cytoarchitecture of each cortical region changes during the protracted developmental period of our study. We stained for the presynaptic vesicular glutamate transporter VGlut2, which labels thalamic terminals in the neocortex (Hur and Zaborszky, 2005). Throughout development, our staining mirrored known thalamic innervation patterns in each region (Fig. 2*A*–*D*). To determine changes in cortical thickness and laminar cytoarchitecture between P5 and P55, we calculated the distance from the pial surface to either the peak of the L3 VGlut2 band, which corresponds to the peak of mediodorsal thalamus innervation in PFC (Collins et al., 2018), or the start of the L4 VGlut2 band, which delineates the top of barrels in SSp (Sehara et al., 2010; Fig. 2). There was a significant increase in these values between P5 and P15 in both regions but little change thereafter (Fig. 2*A*–*D*). In addition, we performed automated counts of DAPI-labeled nuclei across the depth of PFC and SSp. Consistent with the cortical expansion observed in our VGlut2 data and known periods of developmental apoptosis in the neocortex (Wong and Marín, 2019), we observed a significant reduction in cell density at all cortical depths between P5 and P15, after which values stabilized (Fig. S2). Using these data and previously published values for cortical layers in the adult (see Materials and Methods), we were able to assign PSD95 and SAP102 labeling to individual layers across development. Our ability to define thalamo-recipient layers across development allowed us to assess if PFC and SSp regions develop in a thalamus-first manner, or not.

### Distinct layer- and region-specific development in SSp and PFC

We first focused our analysis on SSp, whose development is layer-specific and whose sensory critical periods terminate around P20 (Lendvai et al., 2000; Stern et al., 2001; Erzurumlu and Gaspar, 2012). At the level of raw fluorescence, both PSD95 and SAP102 expression showed a significant developmental increase across the different layers of SSp (Fig. 3*A*,*B*; two-way ANOVA; PSD95 layer *p* ≤ 0.0001; age *p* ≤ 0.0001; SAP102 layer *p* ≤ 0.0001; age *p* ≤ 0.0001). To analyze the relative trajectory of expression in each layer, we took the individual fluorescence traces, which represent the distribution of PSD95 and SAP102 across cortical layers, and peak normalized them to the largest intensity value in each trace. This normalization allowed us to directly compare the laminar distribution of PSD95 and SAP102 at different ages (Fig. 3*C*,*D*) while accounting for changes in the total PSD95 expression across development (Fig. 3*A*,*B*). To control for differences in layer thickness, both between layers and across development, we also calculated the normalized labeling density, which takes the average normalized intensity values and divides them by the thickness of each layer at each age. This allows us to assess how enriched PSD95 and SAP102 are in individual layers at different ages. At P5, we observed enrichment of PSD95 and SAP102 staining in granular and infragranular layers that overlap with observed VGlut2 staining patterns (Fig. 2), suggesting early synapse maturation in layers associated with nascent thalamic innervation (Daw et al., 2007; Marques-Smith et al., 2016). For PSD95, we observed that this initial bias toward thalamo-recipient layers decreased over development, with a significant upregulation of relative PSD95 labeling in supragranular layers throughout adolescence (Fig. 3*C*), such that by adulthood the greatest density of PSD95 signal was in L1 and L2/3 (two-way ANOVA. PSD95 layer *p* ≤ 0.0001; age *p* = 0.028). SAP102 levels were enriched within L4 of SSp at all ages (Fig. 3*D*) with no developmental shift in their distribution (two-way ANOVA; SAP102 layer *p* ≤ 0.0001; age *p* = 0.79), in marked contrast to the adolescent upregulation of PSD95 observed in superficial layers. These findings are consistent with thalamus-first development and also highlight how feedforward (thalamic) and feedback (L1) synapses mature at different rates in SSp.

In PFC, PSD95 and SAP102 expression also showed a significant developmental increase across individual layers (Fig. 4*A*,*B*; two-way ANOVA; PSD95 layer *p* ≤ 0.0001; age *p* ≤ 0.0001; SAP102 layer *p* ≤ 0.0001; age *p* = 0.001). We again peak normalized the synaptic protein distributions and calculated the normalized labeling density to directly compare their laminar profiles across development (Fig. 4*C*,*D*). As in SSp, PSD95 labeling density varied by layer, showing enrichment in thalamo-recipient L1 and L3 and lower levels in L6 (Fig. 4*C*,*D*). Surprisingly, the distribution of PSD95 intensity was remarkably consistent between layers, with no significant effect of age (two-way ANOVA; PSD95 layer *p* ≤ 0.0001; age *p* = 0.28). SAP102 labeling showed a distinct laminar profile, being greatest in L2–5 and least in L1 and L6 with again no change across development (two-way ANOVA; SAP102 layer *p* ≤ 0.0001; age *p* = 0.75). These data suggest that although PFC undergoes significant glutamatergic synapse maturation during the first months of postnatal life, this occurs in a layer-independent rather than a “thalamus-first” manner.

### Adolescent changes in PSD95 levels vary across the cortical hierarchy

The PFC and SSp both showed changes in MAGUK expression across adolescence, but there were clear region-specific differences in their laminar maturation. To extend these findings, we next sought to determine how other cortical regions across the hierarchy develop. To do so we, performed whole-brain imaging of PSD95 and SAP102 pre- (P25) and post-adolescence (P55; Fig. 5*A*). Our hypothesis was that layer-invariant maturation would feature in cortical regions toward the top of the hierarchy, while the delayed maturation of supragranular layers observed in SSp would predominate in lower-order cortices. At each age, we aligned images to the Allen Brain Atlas analyzing 18 regions spanning the cortical hierarchy (Fig. 5*A*,*B*; Harris et al., 2019). The hierarchical position for each brain region was based on previously described analysis that incorporates cell-type–specific axon projections from a given source region, combining corticocortical (CC), thalamocortical (TC), and corticothalamic (CT) projections. The regional hierarchy is determined based on the proportion of connections between given brain areas that are determined to be either feedforward (indicating that a region is below another in the hierarchy) or feedback (indicating that a region is above another). The feedforward or feedback nature of a connection is assigned based on the source layer of the neurons mediating the projection and, for cortical projections, the laminar distribution of the axons in the target structure (Harris et al., 2019). By comparing the change in fluorescence intensity between P25 and P55, we were able to determine how the laminar maturation varied in regions with different hierarchical positions and distinct functional roles within the cortical network. We focused our analysis on PSD95 as we observed minimal adolescent change in SAP102 levels (Fig. S3*A*). Calculating the absolute change in PSD95 between P25 (*n* = 9) and P55 (*n* = 11) revealed a broad, cortex-wide increase with age (Fig. 5*C*), which was largest in superficial layers (Fig. S3). The absolute change in PSD95 levels was correlated with the cortical hierarchy in L1 (L1, slope = −4521; *p* = 0.04), but not other layers (L2/3, slope = −2143; *p* = 0.2; L4, slope = 2312; *p* = 0.2; L5, slope = 1280; *p* = 0.3; L6, slope = 397; *p* = 0.7; Fig. 5*D*), consistent with the adolescent increase we observed in superficial layers of SSp.

The absolute change in PSD95 levels indicates how much individual layers change but is not ideal for comparing between structures with different baseline levels of PSD95 expression at P25. To resolve this, we calculated two additional measures. If our hypothesis was correct, in lower-order areas, superficial layers should mature more between P25 and P55 than deep layers, while in higher-order areas, the layers should mature to the same extent. To test this, we calculated the ratio of PSD95 intensity between L6 and other layers, which also controls for interslice labeling variability, and compared this ratio at the two ages (Fig. S3). Importantly, this new dataset confirmed our previous results, with PSD95 expression in the prelimbic PFC maturing in a layer-independent manner, while SSp showed greater supragranular maturation during adolescence (Fig. S3*C*). When correlating the change in L6 ratio against the cortical hierarchy, there was a significant effect for superficial layers L1 (L1, slope = −0.27; *p* = 0.001) and L2/3 (L2/3, slope = −0.11; *p* = 0.04) but not L5 (L5, slope = 0.02; *p* = 0.6). However, when we performed similar analysis for the relative change in PSD95 across adolescence, there was no significant effect across any layer (L1, slope = −0.22; *p* = 0.09; L2/3, slope = −0.01; *p* = 0.9; L4, slope = 0.15; *p* = 0.17; L5, slope = 0.11; *p* = 0.18; L6, slope = 0.05; *p* = 0.56; Fig. 5*E*,*F*).

Although these data suggest that changes in the cortical circuit may be linked to the hierarchy, we observed significant variability at different hierarchical positions. For example, RSPv, which sits toward the middle of the hierarchy, shows little layer specificity while higher-visual areas such as VISa and VISam mature in a layer-specific manner (Fig. 5*C*,*D*; Fig. S3). This led us to ask if the distribution of PSD95 across individual layers, which also varied considerably between brain region (Fig. S4), may inform how individual brain regions change during adolescence. To explore this, we performed unbiased cluster analysis of the raw PSD95 distributions. This revealed two clusters, with Cluster 1 dominated by 12 sensory–motor regions, including primary and higher sensory areas, while Cluster 2 contained six higher-order association cortices (Fig. 6*A*,*B*). The segregation of brain regions into these two clusters was similar at P25 and P55 suggesting they represent a fairly static feature of the connectome (Fig. 6*A*; Fig. S4). Comparing the relative adolescent change in PSD95 levels between the clusters revealed that Cluster 1 displayed a greater increase in L1 (PSD95 P55/P25 ratio, Cluster 1 = 1.62 ± 0.03; Cluster 2 = 1.37 ± 0.03; *p* ≤ 0.0001) and L2/3 (PSD95 P55/P25 ratio, Cluster 1 = 1.49 ± 0.02; Cluster 2 = 1.37 ± 0.04; *p* = 0.018) compared with association regions in Cluster 2 (Fig. 6*C*), an effect not observed when comparing deep layers (Fig. S4). This suggests that function, rather than hierarchy, may be the main driver of the observed differences across regions. The hierarchical effects we observe may instead be explained by the enrichment of association cortices toward the top of the hierarchy. To confirm this, we performed an analysis restricted to visual areas that span the hierarchy (Fig. 5*B*; Harris et al., 2019). This revealed no correlation in any layer (L1, slope = −31; *p* = 0.99; L2/3, slope = −1159; *p* = 0.63; L4, slope = −148; *p* = 0.98; L5, slope = −55; *p* = 0.98; L6, slope = −983; *p* = 0.54; Fig. 6*D*). These findings suggest that cortical laminar development is strongly linked to function rather than simply position in the cortical hierarchy. Interestingly, differences in synaptic properties in the two clusters are already present at P25 (Fig. 6*A*; Fig. S4) and are further strengthened via the process of superficial layer synapse maturation during adolescence (Fig. 6*B*; Fig. S4) suggesting that enrichment of L1 synapses in sensory–motor regions is a core feature of their functional connectome that matures during adolescence.

### Postnatal development of PSD95 synaptic puncta

**Figure 7.**
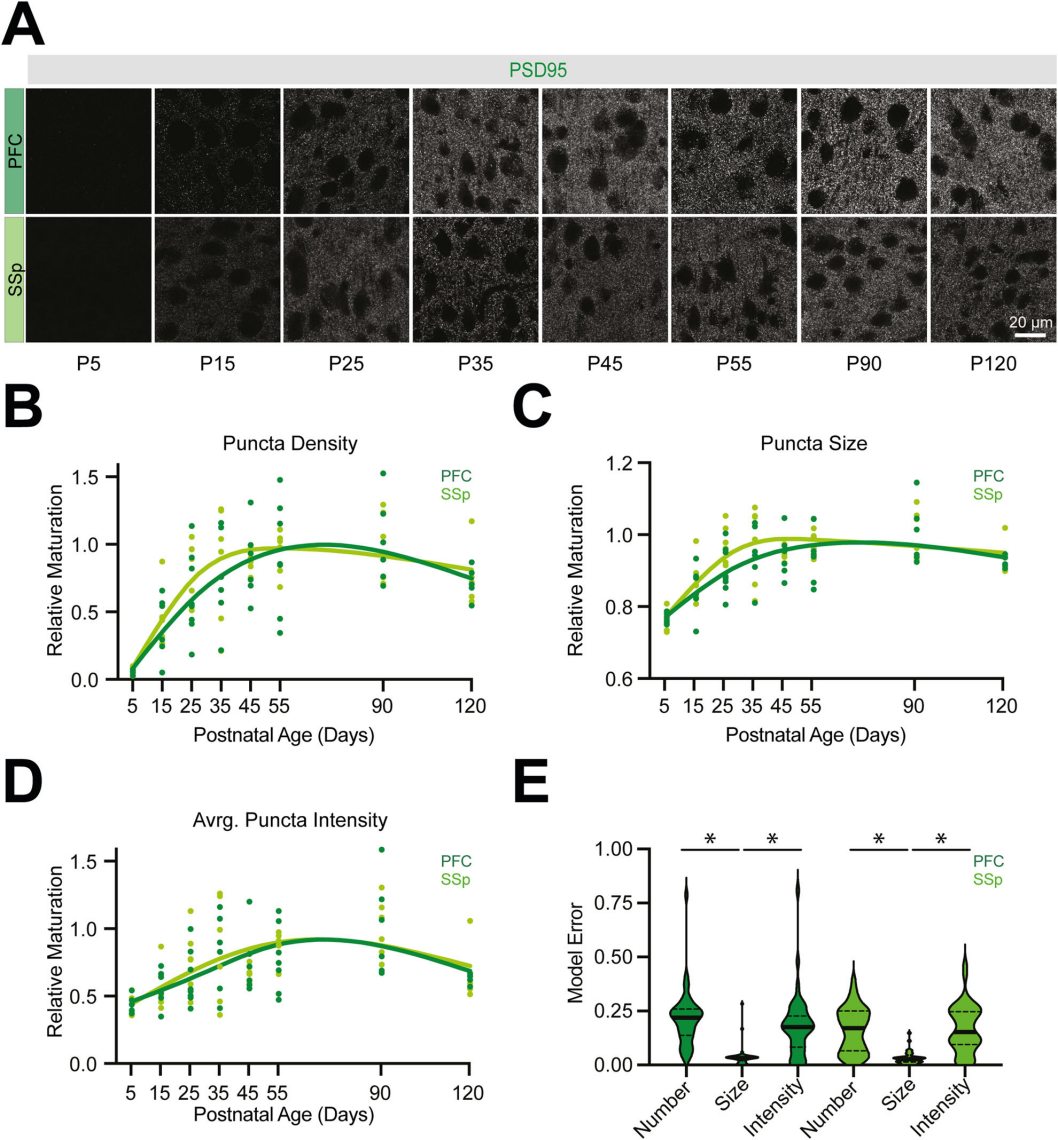
***A***, Representative high-magnification images showing example PSD95 puncta in PFC (top) and SSp (bottom) at different developmental ages. ***B***, Relative maturation of average puncta density in PFC and SSp normalized to adult levels (P90). Points represent the average puncta density across the cortical depth for individual mice with fit to the individual data points. ***C***, As ***B*** but for average puncta size. ***D***, As ***B*** but for average puncta intensity. ***E***, Absolute model error values. The full model combines the number, size, and intensity values to predict relative PSD95 maturation (Fig. S5*E*). Model error represents the absolute change in the prediction after removing either number, size, or intensity data from the model of PFC or SSp development. Large numbers indicate individual parameters contribute a large amount to the prediction and therefore likely contribute significantly to changes in PSD95 maturation, while small values indicate minimal contribution. PFC, number versus size *p* ≤ 0.0001; intensity versus size *p* ≤ 0.0001; number versus intensity *p* = 0.86; SSp, number versus size *p* ≤ 0.0001; intensity versus size *p* ≤ 0.0001; number versus intensity *p* = 0.99. Number of mice per age group, PFC, P5 = 6, P15 = 6, P25 = 8, P35 = 6, P45 = 6, P55 = 7, P90 = 6, P120 = 5; SSp, P5 = 6, P15 = 6, P25 = 6, P35 = 6, P45 = 6, P55 = 6, P90 = 6, P120 = 5. Values are shown as mean average with smoothing spline fit to the individual data points (***B***–***D***) or median with quartiles (***E***). *p < 0.05.

Our analysis up until this point has been performed on bulk fluorescence levels and therefore does not inform about the synaptic mechanisms that drive these changes. Bulk fluorescence signals are a product of the number, size, and fluorescent labeling intensity of individual synaptic puncta. Therefore, to determine how specific synaptic features contribute to the observed developmental changes in PSD95 levels, we performed high-magnification confocal imaging across the cortical depth and applied an automated detection algorithm to segregate individual puncta (Fig. S5). For initial analysis, we pooled data across layers and normalized to the adult values, allowing us to compare with bulk fluorescence data (Fig. 1). At P5, we observed a very small number of puncta in both regions (Fig. 7*A*,*B*). To confirm that this was not due to insufficient excitation of the fluorophore, we also imaged P5 slices at 2× and 2.5× laser power. Although this yielded a slight increase in puncta number, it was still well below levels observed at P15 with the 1× laser power (Fig. S5*D*). Moreover, cortical PSD95 expression is very low during the first postnatal week (Frank et al., 2016) ruling out the possibility of a significant “hidden” pool of PSD95 positive puncta at this time. From P15 onward, we observed pronounced increase in the synapse number in both PFC and SSp, with a similar yet smaller shift in puncta size and mean intensity (Fig. 7*B*–*D*). To determine which puncta parameters best contributed to the development of bulk fluorescence, we modeled the change in PSD95 fluorescence as a product of puncta density, mean intensity, and size (see Materials and Methods). This approach closely approximated the developmental profile of the bulk fluorescence data in both PFC and SSp, as expected (Fig. S5*E*). Comparing the full model to a reduced model where one of the variables was removed revealed that the main contributors to the development of PSD95 signal were changes in puncta number and intensity, with size having a relatively small effect (Fig. 7*E*; median PFC error, number 0.22, size 0.03, intensity 0.17; number vs size *p *≤ 0.0001; intensity vs size *p* ≤ 0.0001; number vs intensity *p* = 0.86; median SSp error, number 0.17; size 0.03, intensity 0.15; number vs size *p* ≤ 0.0001; intensity vs size *p* ≤ 0.0001; number vs intensity *p* = 0.99).

**Figure 8.**
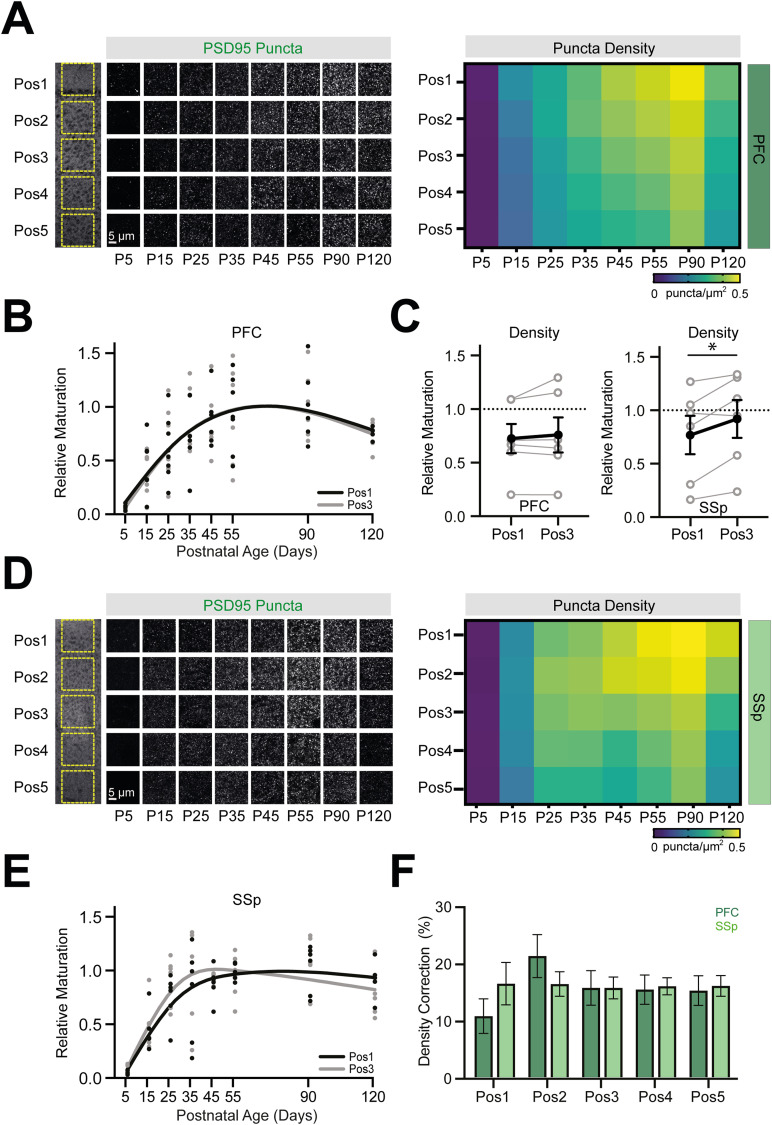
***A***, Left, Representative high-magnification images from the PFC showing PSD95 puncta labeling at Positions 1–5 spanning the cortical depth. Right, Heatmap showing average PSD95 puncta density at each PFC imaging position across development. ***B***, Relative maturation of average puncta density in PFC at Position 1 (Pos1) and Position 3 (Pos3) normalized to adult levels (P90). Points represent the average puncta density for individual mice with smoothing spline fit to the individual data points. ***C***, Left, Relative PFC PSD95 puncta density in Position 1 and Position 3 at P35 showing pairwise, within-slice comparisons. Right, Relative SSp PSD95 puncta density in Position 1 and Position 3 at P35 showing pairwise, within-slice comparisons. PFC *p* = 0.4; SSp *p* = 0.03. ***D***, As ***A*** but for SSp. ***E***, As ***B*** but for SSp. ***F***, Percentage correction in PSD95 puncta density due to nuclear area across the different imaging positions in PFC and SSp. Number of mice per age group: PFC, P5 = 6, P15 = 6, P25 = 8, P35 = 6, P45 = 6, P55 = 7, P90 = 6, P120 = 5; SSp, P5 = 6, P15 = 6, P25 = 6, P35 = 6, P45 = 6, P55 = 6, P90 = 6, P120 = 5. Values are shown as mean average ± SEM, with exception of data in ***B*** and ***E*** which show smoothing spline fit to the individual data points. *p < 0.05.

To assess laminar changes in PSD95 puncta, we imaged across the cortical depth using five equidistant positions (Fig. 8*A*,*D*), with Position 1 closest to the pial surface and Position 5 the white matter (see Fig. S6 for layer alignment). In the adult PFC, the highest synapse density was observed in superficial layers (Fig. 8*A*). Because individual layers have differences in cell density (Fig. S2) and synapses are absent from the area around the cell nucleus (Fig. 6), we performed additional puncta density analysis to account for differences in cell density (see Materials and Methods). In these data, the lack of nuclei in L1 and high density in L2/3 cause a slight shift in the PFC values (Fig. S7), with the biggest increase in puncta density in Position 2, which overlaps with ascending thalamocortical input from the mediodorsal thalamus (Collins et al., 2018). The relative development of PFC was found to be layer-independent, with the maturation of synapse density for superficial (Position 1) and middle layers (Position 3) indistinguishable during adolescence (Fig. 8*B*,*C*; P35 relative maturation PFC Position 1, 0.73 ± 0.14; Position 3, 0.76 ± 0.16; *p* = 0.4).

In SSp, we found that puncta density was highest in middle layers (Position 3) until P35. Subsequent late maturation of superficial layers meant that by adulthood, there was greater synapse density in Positions 1 and 2 (Fig. 8*D*). Consistent with the thalamus-first development observed in SSp, the relative maturation of puncta density in L4 (Position 3), matured earlier than superficial layers (Position 1; Fig. 8*C*–*E*; P35 relative maturation SSp Position 1, 0.77 ± 0.18; Position 3, 0.92 ± 0.18; *p* = 0.03). When correcting for differences in neuronal density we found largely similar results (Fig. 8*F*; Fig. S7). For both regions, we performed similar analysis for the puncta size and mean intensity (Fig. S8). In SSp, puncta size mirrored puncta density, showing a significant bias toward earlier maturation in Position 3, which overlaps with L4 (P35 relative maturation SSp Position 1, 0.95 ± 0.05; Position 3, 0.99 ± 0.04; *p* = 0.006), while mean intensity developed at a similar rate across layers (P35 relative maturation SSp Position 1, 0.8 ± 0.16; Position 3, 0.87 ± 0.17; *p* = 0.15). In contrast, in PFC, both synapse size (P35 relative maturation PFC Position 1, 0.93 ± 0.03; Position 3, 0.95 ± 0.03; *p* = 0.002) and intensity (P35 relative maturation PFC Position 1, 0.78 ± 0.11; Position 3, 0.83 ± 0.12; *p* = 0.02) showed a small yet significant bias toward maturing earlier in deeper layers.

In summary, these findings corroborate our earlier widefield imaging experiments, highlighting key differences in the way that PFC and SSp develop. In the adult, individual layers display notable differences in relative puncta density, with both regions showing enrichment of puncta in supragranular layers, mirroring previous results in adult mice (Zhu et al., 2018). However, individual layers largely develop in unison across PFC, while in SSp, there is a sequential maturation with granular and infragranular layers developing earlier than supragranular layers. Changes in PSD95 levels are driven by an increasing number of synapses that contain more PSD95 at older ages.

### Modeling synapse dynamics in the developing cortex

**Figure 9.**
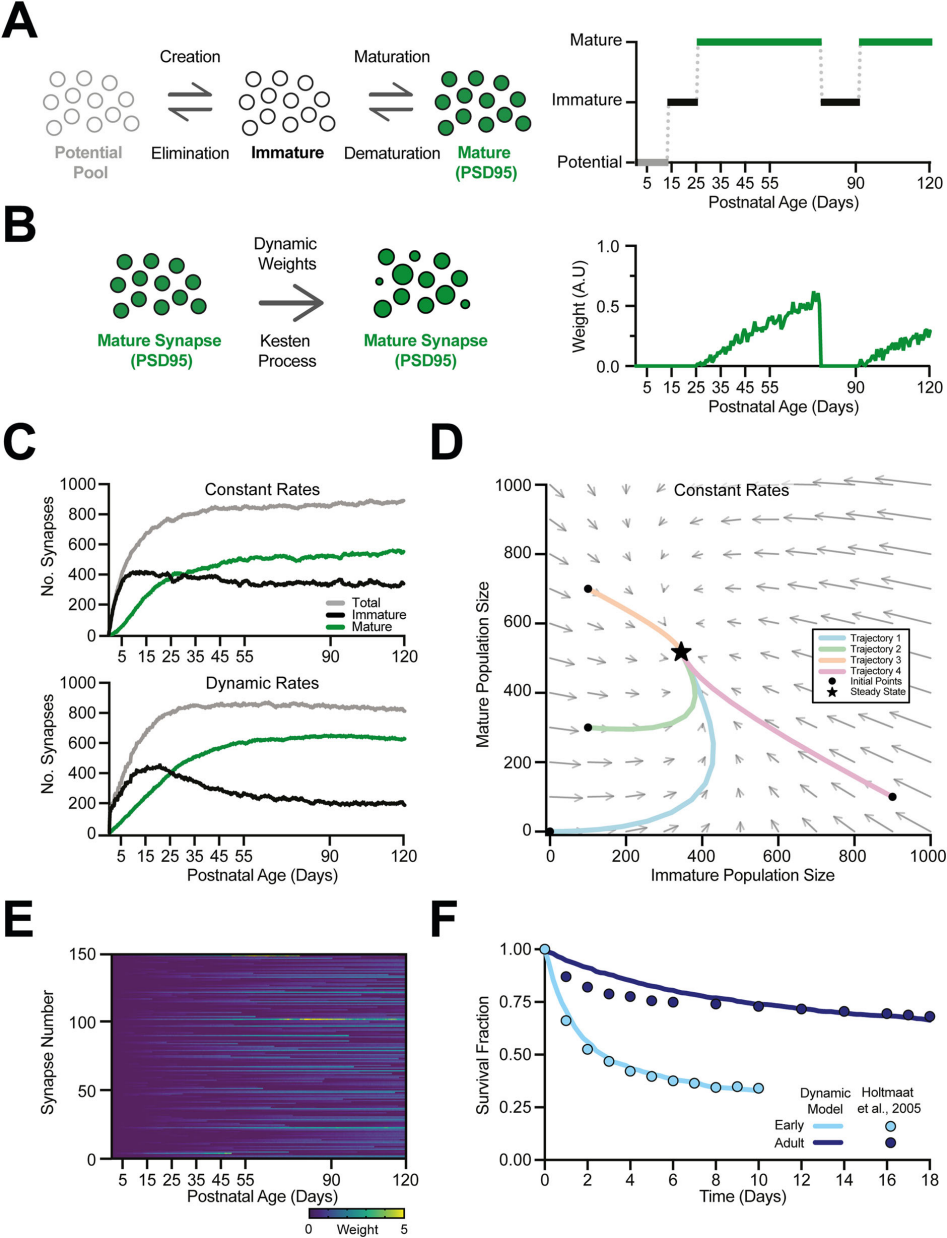
***A***, Left, Model schematic showing how synapses move between potential, immature, and mature populations. Right, Example time series of a single synapse's state during a simulation of 120 postnatal days, showing stochastic transitions between populations across development. ***B***, Left, Schematic showing how the mature synapses' weights are dynamically modulated during development based on a Kesten process (see Materials and Methods). Right, Example synapse weight showing stochastic changes across development, same simulation as panel ***A***. ***C***, Top, Random walks model with constant rates governing the transition between synapse populations across development. Changes in immature, mature, and total synapse number are shown. Bottom, Similar but for model with dynamic weights across development (Fig. S9*B*). ***D***, Phase plot calculated from the differential equation model with constant rates, showing convergence of developmental trajectories in synapse number toward a fixed final state, despite different initial starting conditions. ***E***, Heatmap showing lifetime synapse weight dynamics across development for a representative simulation of a population of 150 individual synapses, showing both persistent and transient synapses across development. ***F***, Survival dynamics of synapses relative to two different reference ages: early (P16) and adult (P70). The plot compares results from the dynamic rates model (solid curves) and experimental data from (Holtmaat et al., 2005; filled circles).

In contrast to previous studies of dendritic spine density (Bourgeois et al., 1994; Pöpplau et al., 2024), we did not observe any adolescent pruning of PSD95 puncta across our analyses. We reasoned that this could be because not all spines, particularly immature ones, contain functional synapses or express PSD95 (Knott et al., 2006; Arellano et al., 2007; Zhu et al., 2018) and that the proportion of immature and mature spines is highly dynamic across development (Holtmaat et al., 2005). Consequently, our PSD95 data may better model the emergence of mature, stable synapses in the developing brain, rather than the total synapse population. To test this hypothesis, we built a computational model of synapse dynamics which incorporated previous in vitro and in vivo studies (see Materials and Methods). In this theoretical model, at each stage of development, individual synapses occupied either a reserve pool of potential synapses, a population of immature synapses, or a population of mature synapses (Fig. 9*A*). We first modeled synapse dynamics using constant rates fixed across development to govern the transition between these populations. We tested two versions of the model: a random walks model and a differential equation model, which yielded similar results (Fig. 9*C*; Fig. S9). Interestingly, we found that, although we were able to generate developmental trajectories for mature synapses that mirrored our PSD95 data using these models (Fig. 9*C*), pruning of the total synapse population was mathematically impossible if rates were fixed across development and the total synapse count begins at zero (see Fig. 9*C* and proof in Text S1). Next, we applied dynamic rates across development (Fig. 9*C*; Fig. S9), consistent with in vivo data on spine dynamics in the postnatal neocortex that show how the rate of creation and elimination decreases with age (Holtmaat et al., 2005). Under these conditions, the three synapse populations in our model displayed distinct trajectories during development. The immature population peaked early (<P25) before decaying to reach a stable level in adulthood. The mature population mirrored our observed developmental profile of PSD95, displaying a slow monotonic increase before stabilizing toward the end of adolescence. Finally, the total synapse population peaked prior to adolescence, before undergoing a developmental reduction (Fig. 9*C*).

When synapses reached the mature population, their weight was also dynamically adjusted (Fig. 9*B*), with larger synapses resistant to dematuration (Knott et al., 2006; Taft and Turrigiano, 2014; Fig. S9). We modeled synapse lifetimes across development, observing both short-lived synapse and stable synapses that persisted across development (Fig. 9*E*). By computing the survival fraction for synapses at early (P16) and adult (P70) developmental ages, we were able to show that immature synapses were less stable than adult synapses (Fig. 9*F*), mirroring previous in vivo work (Holtmaat et al., 2005). These modeling results highlight potential mechanisms that govern synapse dynamics in the developing cortex. To determine how individual model parameters influence the properties of this system, we performed sensitivity analysis. This revealed a stark separation in the developmental roles of each part of the model. The rates of synapse creation and elimination govern the developmental age at which the peak of the different synapse populations occurs, while the maturation and dematuration rates govern the final number of mature and immature synapses (Fig. S9). Overall, these modeling results reconcile prior findings with our new data, showing that both developmental pruning of total dendritic spine number and a monotonic increase in mature (putative PSD95+) synapses can coexist in the developing neocortex.

## Discussion

This study maps glutamatergic synapse development across layers and regions of the mouse neocortical hierarchy by quantifying changes in fluorescently labeled MAGUK proteins PSD95 and SAP102. We find key differences in PSD95 expression between primary sensory and prefrontal areas, with sensory cortex development occurring earlier and in a layer-specific manner. In contrast, prefrontal development occurs later and is largely synchronous across layers. This relationship is restricted to PSD95, with SAP102 displaying earlier expression and few layer-dependent effects. Adolescence is important for PFC maturation, and we observe pronounced changes in PFC synapses across all layers during this time. However, in SSp, we also observe maturation in superficial synapses, particularly in L1. Cortical L1 is a key locus for the integration of top–down inputs (Schuman et al., 2021; Huang et al., 2024), such that our findings are consistent with a model whereby feedforward pathways develop early, followed by the maturation of higher-order areas and their feedback projections, culminating in the final stage of outside–in development. Importantly, we find that this change occurs across much of the cortex, suggesting adolescence represents an important period for the development of top–down inputs.

Adolescence is a key time for the PFC, which undergoes changes in connectivity, receptor expression, and intrinsic physiology, resulting in refinements to network dynamics and cognition (Larsen and Luna, 2018; Delevich et al., 2018; Anastasiades et al., 2022; Pöpplau et al., 2024; Zhu et al., 2024). Our findings are consistent with the idea that PFC synapses increase the relative occupancy and amount of PSD95 synaptic protein during adolescence. This likely helps stabilize synapses (Ehrlich et al., 2007; Delevich et al., 2018; Bulovaite et al., 2022) while also providing a scaffold for key components of the synaptic signaling machinery (Sheng and Kim, 2011; Lohmann and Kessels, 2014). Our findings suggest that this process occurs at the same time across layers. However, each layer contains many cell types, and although certain inputs are biased toward a given layer, they may target specific neurons across distinct parts of their dendritic arbor (Anastasiades and Carter, 2021). This is also true in L1, with previous studies highlighting cell-type–specific connectivity (Anastasiades et al., 2021), developmental dynamics, and plasticity in this layer (Ibrahim et al., 2021; Klappenbach et al., 2025). Our findings are therefore not inconsistent with input- or cell-type–specific effects (Johnson et al., 2016; Pattwell et al., 2016), although it appears that, at least at the level of layers, such a process is less evident in PFC than in sensory and motor areas.

The protracted maturation of L1 is consistent with the cortex gradually transitioning from a feedforward-dominated network to one where feedback projections take on greater prominence. This phenomenon was recently reported in human in vivo studies (Pines et al., 2023), with increased feedback marking an inflection point at which the brain transitions to adolescence (Dong et al., 2021). This occurs contemporaneously with the maturation of both the cortical neurons providing feedback (Nabel et al., 2020) and the higher-order thalamocortical system (Sydnor et al., 2025). Top–down connections have increasingly been incorporated into brain-inspired machine learning models that use feedback as a teaching signal (Sacramento et al., 2018; Tugsbayar et al., 2025). In such models, it is often advantageous to use a dynamic, or adaptive, learning rate to help optimize the network. It is interesting to speculate that the brain may engage a similar strategy, employing increasingly sophisticated internal predictions and outcome monitoring to guide learning as development ensues (Hartley and Somerville, 2015; Buzzell et al., 2017). Consistent with this idea, L1 is a key site of integration for top–down multimodal signals (Schuman et al., 2021; Huang et al., 2024) that help regulate learning, sensory encoding, perceptual decision-making, and flexible behavior (Letzkus et al., 2011; Williams and Holtmaat, 2019; Banerjee et al., 2020; Doron et al., 2020; Furutachi et al., 2024; Mo et al., 2024). Importantly, weak top–down signaling is observed in schizophrenia, a neurodevelopmental disorder that emerges during adolescence and whose symptomatology includes impairments in learning and decision-making (Lyu et al., 2025). L1 maturation may therefore represent a key locus for the emergence of flexible cognition and a key site of vulnerability in adolescent mental health disorders.

The distinct developmental timings of SSp and PFC are consistent with hierarchical maturation (Gogtay et al., 2004; Pinto et al., 2013; Reh et al., 2020). However, evidence from primates shows synaptogenesis occurring concurrently throughout the cortex, with synapse density peaking prior to puberty, before undergoing adolescent pruning (Zecevic and Rakic, 1991; Bourgeois et al., 1994). Plasticity in SSp is also observed in nongranular layers during adolescence but less so thereafter (Fox, 2002). Although our data support the hierarchical development of the cortex, we see adolescent changes in PSD95 levels across the neocortex. We also observe increased PSD95 positive puncta, consistent with previous studies (Cizeron et al., 2020). A similar disconnect between synapse number and synaptic protein expression has been reported for frontal regions across species, with synapse elimination observed in adolescent humans (Huttenlocher and Dabholkar, 1997), primates (Bourgeois et al., 1994), and rodents (Pöpplau et al., 2024) despite bulk proteome studies displaying a steady increase in levels of PSD95 and other synaptic markers (Glantz et al., 2007; Webster et al., 2011; Pinto et al., 2013). Our computational modeling provides a theoretical framework to reconcile these data whereby nascent, immature synapses, which typically lack or express low levels of PSD95, are produced at high rates early in development, peaking prior to adolescence. Dynamic shifts in spine formation and elimination cause a subsequent reduction in spine number (Holtmaat et al., 2005), while at the same time mature synapses incorporate PSD95 clusters of increasing size. This occurs concomitantly with the maturation of synaptic molecular diversity, which also stabilizes toward the end of adolescence (Cizeron et al., 2020).

Integrating these data, we can explain changes in glutamatergic synapses development across the rodent neocortex. PSD95 is associated with glutamatergic spines; however, the two are not synonymous, spines form even after complete loss of PSD95 (Yusifov et al., 2021), PSD95 may be present on aspiny GABAergic interneurons (Kawabata et al., 2012), and spines can be enriched for distinct MAGUK proteins (Zhu et al., 2018). This process is also highly developmentally regulated. The early neocortex is dominated by immature “silent” synapses which primarily signal via NR2B subunits of the NMDA-R (Hanse et al., 2013) which couples to SAP102 to promote synapse formation (Chen et al., 2011). This is consistent with higher levels of SAP102 and very low levels of PSD95 in both PFC and SSp prior to P15 and observations that the early synaptic maturation of these brain regions is similar (Myme et al., 2003). At P12, the brain undergoes a sensory awakening following eye opening, active whisking, and the unblocking of the auditory canal (Wu et al., 2024). This drives rapid localization of PSD95 to the synapse (Yoshii et al., 2003), which we observe to increase at P15. This shift facilitates synapse maturation via activity dependent AMPA-R insertion and the transition to NR2A-mediated NMDA-R signaling (Elias et al., 2008; Hanse et al., 2013). Over time this causes an abundance of nascent synapses, which peak in the days following (Holtmaat et al., 2005). As development ensues, synapses continue to incorporate PSD95, while a subset of synapses increase their size and stability. Synapses that fail to undergo stabilization are lost (Holtmaat et al., 2005; Knott et al., 2006). This process likely contributes to the termination of developmental plasticity (Huang et al., 2015).

Our modeling of spine dynamics replicates the adolescent changes in PSD95 puncta we observe as well as in vivo observations (Holtmaat et al., 2005). Importantly, these effects do not appear to be driven by differences in the rate at which spines are formed in different layers or neuronal subcellular compartments (Romand et al., 2011; Tjia et al., 2017). Our theoretical model instead suggests that maturing PSD95 puncta may act as a “developmental clock” tracking the relative maturity and stability of synapses. Although various biological mechanisms can control the rates of synapse elimination, maturation, and dematuration (Yoshihara et al., 2009), it remains unclear exactly how these rates may vary across brain regions; differences in neural activity (Yi Zuo et al., 2005), synaptic/structural proteins (Agoglia et al., 2017; Zhu et al., 2018), cytoskeletal dynamics (Honkura et al., 2008), sex hormone receptors (McEwen and Milner, 2017; Delevich et al., 2018), or microglial pruning (Schalbetter et al., 2022; Pöpplau et al., 2024) may fine-tune the rate at which synapses develop to yield region and layer-specific trajectories. It will be important to determine the mechanisms behind this processes given significant differences exists, even within a single brain region.

Another key finding is the difference in adolescent laminar development between brain regions. Although we observe a loose correlation between L1 adolescent synapse maturation and the cortical hierarchy, our analysis implies that the effect is more likely driven by function, rather than hierarchical position per se. Indeed, when solely analyzing visual cortices, we see no effect of hierarchy. Consequently, the observed effect likely occurs because association cortices are biased toward the top and primary sensory areas toward the bottom of the hierarchy. Why might this laminar difference between sensory–motor and association cortices exist? Our data indicate that enrichment of L1 synapses in sensory–motor areas is present prior to adolescence and is further strengthened during subsequent development. This circuit motif may be particularly important for providing rapid feedback to sensory–motor areas to support learning (Cichon and Gan, 2015; Doron et al., 2020; Benezra et al., 2024). In contrast, learning-related inputs to higher-order associative cortices are more segregated across layers (Anastasiades and Carter, 2021). Alternatively, association cortices may rely more heavily on diffuse neuromodulatory systems, such as dopamine, to guide learning (Glimcher, 2011; Puig and Miller, 2012). Finally, there is evidence that NR2B subunits have a more prominent role in frontal cortices, including their adolescent upregulation in apical dendrites (Flores-Barrera et al., 2014; Bessières et al., 2024). With a different complement of NMDA-R subunits, it is possible L1 synapses retain more plasticity into adulthood or that adolescent changes in association cortices involve distinct mechanisms. Future research is needed to explore these alternative hypotheses.

In summary, adolescence is a key period for the maturation of synapses associated with higher-order circuits in the neocortex, including all layers of association regions and L1 of sensory–motor regions. These changes occur due to increases in the number of synapses incorporating PSD95 molecules and associated increases in synapse strength and stability. Our current study only focuses on individual synapses, without identifying their source or postsynaptic target. In the future, understanding the mechanisms and specificity that governs how these circuits mature will advance our understanding of healthy aging and may be of clinical significance given adolescence is a key period for the emergence of various mental health conditions (Blakemore, 2019; Solmi et al., 2021).
